# Genome analysis of the yeast *Diutina catenulata*, a member of the Debaryomycetaceae/Metschnikowiaceae (CTG-Ser) clade

**DOI:** 10.1371/journal.pone.0198957

**Published:** 2018-06-26

**Authors:** Caoimhe E. O’Brien, Charley G. P. McCarthy, Annie E. Walshe, Dennis R. Shaw, Deirdre A. Sumski, Tadeusz Krassowski, David A. Fitzpatrick, Geraldine Butler

**Affiliations:** 1 School of Biomolecular and Biomedical Science, Conway Institute, University College Dublin, Belfield, Dublin, Ireland; 2 Department of Biology, Genome Evolution Laboratory, Maynooth University, Maynooth, Co. Kildare, Ireland; 3 School of Medicine, Conway Institute, University College Dublin, Belfield, Dublin, Ireland; University of Strasbourg, FRANCE

## Abstract

*Diutina catenulata* (*Candida catenulata*) is an ascomycetous yeast that has been isolated from humans, animals and environmental sources. The species is a contaminant of dairy products, and has been linked to superficial and invasive infections in both humans and animals. Previous phylogenetic analyses have assigned the species to the Saccharomycetales, but failed to identify its specific clade. Here, we report the genome sequence of an environmental isolate of *D*. *catenulata*. Examination of the tRNA repertoire and coding potential of this species shows that it translates the CUG codon as serine and not leucine. In addition, two phylogenetic analyses using 204 ubiquitous gene family alignments and 3,826 single-copy genes both confirm the placement of the species in the Debaryomycetaceae/Metschnikowiaceae, or CTG-Ser clade. The sequenced isolate contains an *MTL*α idiomorph. However, unlike most *MTL* loci in related species, poly (A) polymerase (PAP) is not adjacent to *MTL*α1.

## Introduction

*Candida catenulata* is an ascomycetous yeast, commonly associated with dairy products such as milk [1] and cheese [2,3], including in Ireland [4]. The species has also been isolated from the microbiota of the oral cavity of female canines [5], and from the gastrointestinal tract and the feces of poultry [6], wild birds [6,7,8] and piglets [9]. Its environmental niche is not known, although it has been identified in rural dust [10] and in the estuary of the river Tagus [11]. *C*. *catenulata* is not usually associated with disease in humans. However, rare cases have been described, including in a cancer patient [12] and in vulvovaginal infections [13]. There are suggestions that *C*. *catenulata* could be used in bioremediation because of its ability to degrade hydrocarbons [14].

The phylogenetic relationship of *C*. *catenulata* to other yeast species is unclear. Lachance et al [15] showed that the species forms a clade with *Candida rugosa* and *Candida scorzettiae*, but the authors were unable to determine the exact position on the fungal tree of life. Additional species were added to the clade following the identification of *Candida ranongenesis* from estuarine water, and *Candida mesorugosa*, *Candida neorugosa* and *Candida pseudorugosa* from clinical samples [16,17]. Khunnamwong et al. [18] carried out a detailed phylogenetic analysis of the clade following the identification of three other related endophytic yeasts from rice tissue. Using ribosomal DNA sequencing, they confirmed that all these species are members of the Saccharomycetales, but again, were unable to completely determine the phylogenetic position. Because of new rules on naming yeast species which state that the name *Candida* should only be used for asexual species closely related to *Candida tropicalis* [19], Khunnamwong et al. [18] suggested that the entire clade be renamed as genus *Diutina*, and we follow this proposal here.

As part of an undergraduate research project using an approach similar to that described by Sylvester et al. [20], we isolated *Diutina catenulata* from soil. We generated a draft genome sequence of one isolate and used it to build robust phylogenetic trees. These show that *D*. *catenulata* belongs to the Debaryomycetaceae/Metschnikowiaceae family, and lies outside the Lodderomyces clade. Analysis of tRNA sequences supports the hypothesis that *D*. *catenulata* translates CUG as serine, similar to other species in the Debaryomycetaceae/Metschnikowiaceae.

## Materials and methods

### Strain isolation

*D*. *catenulata* isolate WY3-10-4 was identified from approximately 5 g of soil collected in 15 ml sterile plastic tubes from a canal bank edge near Castleknock, Dublin (GPS co-ordinates 53.381793, -6.370725) in 2016, and isolate UCD133 from soil on a forest path in the Phoenix Park, Dublin (GPS co-ordinates 53.354500,-6.371346) in 2017. No endangered or protected species or locations were involved. Microorganisms were extracted from one spatula of soil was inoculated at 30°C in 9 ml YPD (1% yeast extract, 2% peptone, 2% dextrose) containing 0.03 mg/ml chloramphenicol and 0.1 mg/ml ampicillin in 15 ml plastic screw top tubes for 48 h. 10 **μl** was subcultured to fresh media for 24 h, and then dilutions were plated on YPD agar. The internal transcribed spacer (ITS) region of the rDNA locus was amplified directly from the yeast colonies by PCR using MyTaq Red (Bioline) and primers ITS1 and ITS4 [21]. The PCR products were purified using the NucleoSpin Gel and PCR Cleanup Kit (Machery-Nagel), and sequenced using the same primers by Eurofins Genomics.

### Genome sequencing and assembly

Genomic DNA was extracted from *D*. *catenulata* WY3-10-4 using phenol-chloroform and sequenced on two lanes of an Illumina HiSeq2500, producing 2x250bp paired-end reads. Library preparation and sequencing were carried out at the Earlham Institute, Norwich, UK, using the LITE method (Low Input Transposase-Enabled), a custom Nextera-based system.

Illumina sequencing produced 4,958,140 raw paired-end reads. Low quality bases and adapters were removed using Skewer (v0.2.2) [22] with parameters -m pe (paired end mode) -l 35 (minimum read length allowed after trimming) -q 30 (trim 3’ end of read until quality of 30 or greater is reached) -Q 30 (the lowest mean quality value allowed after trimming) -r 0.001 (maximum allowed error rate) -t 2 (number of concurrent threads). A second round of adapter removal was carried out using TrimGalore (v0.4.3) (https://www.bioinformatics.babraham.ac.uk/projects/trim_galore/) with parameters—paired—length 35 (minimum read length allowed after trimming)—nextera (trim first 12bp of the Nextera adapter)—stringency 3 (length of overlap with adapter sequence required to begin trimming). 3,966,474 high-quality reads were subsequently available for assembly. Trimmed reads were assembled using SPAdes (v3.9.1) [23] with parameters—careful (reduce number of mismatches and short indels in contigs) -t 10 (number of threads) -m 100 (RAM limit in gigabytes). A preliminary analysis suggested that there was a low level of contamination of reads from other species. Contaminating contigs were removed by filtering those less than 1 kb in size, or with k-mer coverage of less than 25% of the average for the assembly. The final assembly consists of 613 contigs, with a total length of 13,099,930 bases and an N50 of 41,233 bp. This includes two rDNA contigs (Dcat_rDNA_01 and Dcat_rDNA_02). A mitochondrial DNA contig (22,336 bp) was also assembled. All data have been deposited in GenBank under Bioproject PRJNA421257. Average assembly coverage was calculated by aligning the trimmed reads used for the assembly against the final assembly with bwa mem (v0.7.15) [24] and the Genome Analysis Toolkit DepthOfCoverage tool (v3.7) was used to calculate the mean coverage across the whole assembly.

Variant analysis was performed to ascertain the ploidy of the isolate. Trimmed reads were aligned against the final assembly sequence with bwa mem (v0.7.15). Duplicate reads were marked with PicardTools MarkDuplicates (v2.8.3) (http://broadinstitute.github.io/picard) and variants called with the Genome Analysis Toolkit HaplotypeCaller (v3.7) [25].

### Phylogenetic analysis

Predicted protein sets for 40 Saccharomycotina species and the outgroup species *Neurospora crassa* were obtained from public databases, and a predicted protein set for *Diutina catenulata* was generated using AUGUSTUS with a *Meyerozyma guilliermondii* training set, generating 7128 predicted genes [26]. Gene family finding was carried out on the 250,403-protein dataset using the random BLASTp approach with an e-value cutoff of 10^−20^ [27,28]. A total of 3,835 single-copy gene families were identified using this approach; of these, 206 families had one ortholog from each species in the dataset. All gene families were aligned using MUSCLE [29], and conserved regions of each alignment were sampled using Gblocks with the default parameters [30]. Nine gene families did not retain character data after sampling and were removed from further analysis. Best-fit evolutionary models were determined for the remaining 3,826 gene families using ProtTest [31]. Maximum-likelihood gene phylogenies were generated for each gene family using PhyML, with 100 bootstrap replicates and each family’s corresponding best-fit model [32].

Heuristic Bayesian supertree reconstruction of 42 species based on 3,826 single-copy gene phylogenies was performed using the ST-RF model as implemented in p4 [33]. Two separate Monte Carlo Markov Chain (MCMC) analyses with 4 chains each were ran for 30,000 generations with *β* = 1, sampling every 20 generations. Both analyses converged after 30,000 generations and a consensus Bayesian supertree phylogeny based on posterior probability of splits was generated from 150 trees sampled after convergence [28,33]. This consensus phylogeny was visualized using iTOL [34] (S1 Fig).

**Fig 1:**
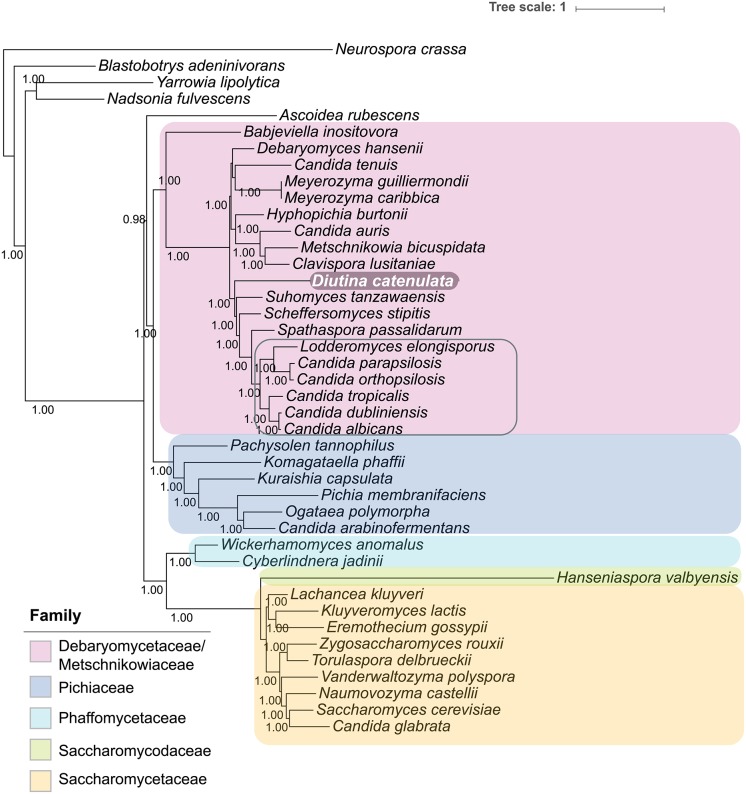
*Diutina catenulata* is a member of the Debaryomycetaceae/Metschnikowiaceae clade. The phylogenetic tree was inferred from a superalignment of 204 ubiquitous gene families from 42 species. A consensus Bayesian supermatrix phylogeny was generated using PhyloBayes [38]. Clades within the Saccharomycotina (Debaryomycetaceae/Metschnikowiaceae, Pichiaceae, Phaffomycetaceae, Saccharomycodaceae and Saccharomycetaceae) are highlighted in color. Species within the Lodderomyces clade in the Debaryomycetaceae/Metschnikowiaceae are surrounded with a gray box. The exact definition of the Lodderomyces clade is not clear, and it may include *Spathaspora* species [48]. The branch supports show Bayesian Posterior Probabilities.

204 ubiquitous gene family alignments retained character data after sampling in Gblocks. From these 204 alignments, a 93,825-character superalignment for 42 species was constructed using FASConCAT [35]. 32,988 phylogenetically-uninformative sites were removed from the superalignment using PAUP*, for a final superalignment length of 60,837 characters [36]. MCMC Bayesian supermatrix reconstruction was performed on the superalignment using PhyloBayes MPI with the default CAT+GTR evolutionary model [37], running two simultaneous chains for 100,000 iterations and sampling every 100 iterations [38]. After the chains converged, a consensus Bayesian supermatrix phylogeny was generated using a burn-in of 1000 trees and the phylogeny was visualized using iTOL (Fig 1).

Alignments and phylogenetic analysis of PAP proteins were implemented in SeaView [39].

### CUG codon analysis

Non-overlapping open reading frames of at least 60 amino acids were predicted using a Python script. The nucleotide sequence of each ORF was translated using the standard genetic code and aligned against a database of proteins from 36 species in YGOB [40,41] using BLASTP with a threshold e-value of 1e-10. Each possible translation of each codon was then assigned a score as follows: for every codon in a predicted ORF, if it aligned with at least 5 proteins in the database, and more than 80% of these had the same amino acid at this site, we assigned a score of 1/n, where n is the total number of proteins aligned. The scores for each codon were then added across all ORFs in the genome. For each codon, the correct translation was identified as the highest scoring translation across all ORFs. The full analysis is shown in S1 File.

### Sequencing of MTL locus

The region surrounding *MTL*α1 was amplified (primers GAAAATGCTATGAGGTCGGG and GACCTGAATTTGCCGTGCTT) from genomic DNA extracted using phenol-chloroform from two *D*. *catenulata* isolates (WY3-10-4 and UCD133). The PCR products were purified using the Macherey-Nagel NucleoSpin Gel and PCR Clean-up Kit and sequenced using the Eurofins Genomics Mix2Seq platform.

## Results and discussion

*Diutina catenulata* was isolated from soil in Dublin, Ireland, by culturing in glucose media and reduced oxygen as described by Sylvester et al [20]. Isolates were identified by sequencing the ITS region, which, for WY3-10-4, differed by only one base from the *D*. *catenulata* type sequence over 385 bases [42]. Phylogenetic analysis by Khunnamwong et al [18] suggested that *D*. *catenulata* lies in an unaffiliated clade within the Saccharomycetales, and is possibly related to the Debaryomycetaceae/Metschnikowiaceae. However, their phylogenetic reconstruction failed to support a single origin of the Debaryomycetaceae/Metschnikowiaceae, and the authors were reluctant to place the species within this family.

Genera such as *Debaryomyces* and *Lodderomyces* are assigned to the family Debaryomycetaceae, whereas the family Metschnikowiaceae is named from the *Metschnikowia* genus, which includes *Metschnikowia bicuspidata*, a pathogen of brine shrimp, as well as a group of large-spored species [43,44,45]. Recent analysis based on whole genome sequences has shown that the Metschnikowiaceae and the Debaryomycetaceae (and possibly also the Cephaloascaceae) form a single, monophyletic clade [46,47,48] (Fig 1). The species in the clade translate CUG as serine, rather than leucine, and are often referred to as the CTG or CUG clade [27,49,50]. It has recently been shown that the yeast *Pachysolen tannophilus* (a member of the Pichiacea, a sister clade to the Debaryomycetaceae/Metschnikowiaceae) also has a non-standard translation of CUG, but in this species CUG encodes alanine rather than serine or leucine [50,51]. It is therefore more accurate to refer to the Debaryomycetaceae/Metschnikowiaceae as the CTG-Ser clade.

To help solve the phylogenetic position of *D*. *catenulata*, we used Illumina technology to generate a draft genome of the WY3-10-4 isolate. A draft genome was assembled with approximately 61.2X coverage. Variant calling against the final assembly identified 928 single nucleotide polymorphisms (SNPs). This lack of variation strongly suggests that the isolate is a haploid. We annotated the genome using Augustus [26], and identified gene families shared with 40 Saccharomycotina species and the outgroup species *Neurospora crassa*.

A superaligment of 204 ubiquitous gene families was used to construct a consensus Bayesian supermatrix phylogeny (Fig 1). The phylogenetic reconstruction matches previous trees constructed from whole genome data [46,47]. Many of the major groups are recapitulated, including the Saccharomycetaceae, Saccharomycodaceae, Phaffomycetaceae, Pichiaceae and the Metschnikowiaceae (Fig 1). *D*. *catenulata* is placed within the Debaryomycetaceae/Metschnikowiaceae with a Bayesian Posterior Probability (BPP) of 1 (Fig 1).

*D*. *catenulata* is found as an outgroup to the Lodderomyces clade, and to *Scheffersomyces*, *Spathaspora* and *Suhomyces*. The phylogenetic placement has strong outgroup support (BPP 1). This conclusion is further supported by a supertree generated from 3,826 single-copy gene phylogenies, which places *D*. *catenula* in the same phylogenetic position (S1 Fig). However, *D*. *catenulata* lies on a long branch in Fig 1, supporting the suggestion by Khunnamwong et al [18] that the entire *Diutina* clade is quite divergent from other characterised species in the Debaryomycetaceae/Metschnikowiaceae.

### Analysis of coding potential of *D*. *catenulata*

**Fig 2:**
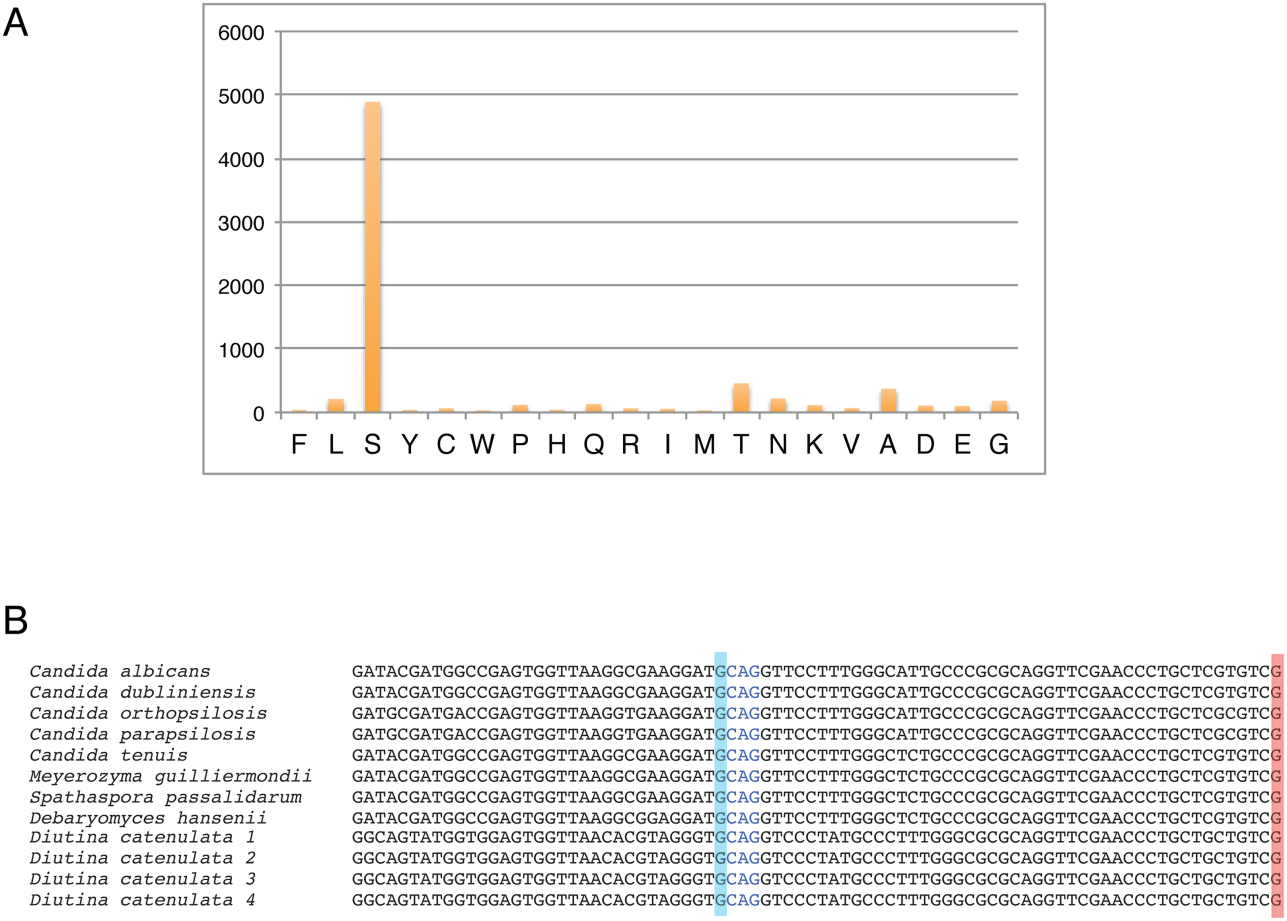
*D*. *catenulata* translates CUG codons as serine. A. Bar plot showing frequencies of amino acid matches to CUG codons in the *D*. *catenulata* genome. The values on the Y-axis represent the number of CUG codon sites that align with the amino acid residues shown on the X-axis in the YGOB protein database [41]. Analysis of all codons is shown in S1 File. B. Comparison of tRNA^Ser^(CAG) from *D*. *catenulata* with the same tRNA encoded by other species in the Debaryomycetaceae/Metschnikowiaceae. The discriminator base at the 3’ end (highlighted in red) is G in tRNA^Ser^(CAG), and A in most tRNA^Leu^(CAG) molecules. The G base just 5’ to the anticodon (highlighted in blue) also reduces leucylation [54].

If *D*. *catenulata* is a member of the CTG-Ser clade, we expect that it translates CUG as serine. To test this hypothesis, we used a bioinformatics method similar to Riley et al [50]. Each *D*. *catenulata* gene was used as a query for a BLAST search against the YGOB database of proteins from 36 yeast species that use the universal genetic code [41]. For each codon site in a *D*. *catenulata* gene, we tabulated the amino acid(s) in other species to which the site was aligned in the BLAST output. The results for each of the 61 sense codons were then summed across all genes. For all codons except CUG, the most common amino acid in alignments was its universal translation, for example AUG codons aligned most commonly with methionine residues. However, for CUG, approximately 4900 CUG sites in *D*. *catenulata* genes aligned with serine in other species, and only 212 with leucine (Fig 2A). This result indicates that CUG is likely translated as serine in *D*. *catenulata*.

Species in the CTG clade translate the codon CUG using a novel tRNA, tRNA^Ser^(CAG), which evolved from an existing tRNA^Ser^ [50,51,52]. We therefore examined the tRNA repertoire of the *D*. *catenulata* genome using tRNAscan-SE [53]. The genome is predicted to encode 285 tRNAs, including 22 tRNA^Leu^ and 20 tRNA^Ser^ molecules. Four identical tRNAs with the anticodon CAG are predicted to translate CUG as serine, and not leucine (Fig 2B). These tRNAs have a G at position 82, the discriminator base, which is characteristic of tRNA^Ser^(CAG) in the Debaryomycetaceae/Metschnikowiaceae; tRNA^Leu^(CAG) molecules have an A at this position [50]. The tRNAs also have a G at base 33, immediately 5’ to the anticodon, which reduces leucylation (Fig 2B) [54].

### MAT locus of *D*. *catenulata*

**Fig 3:**
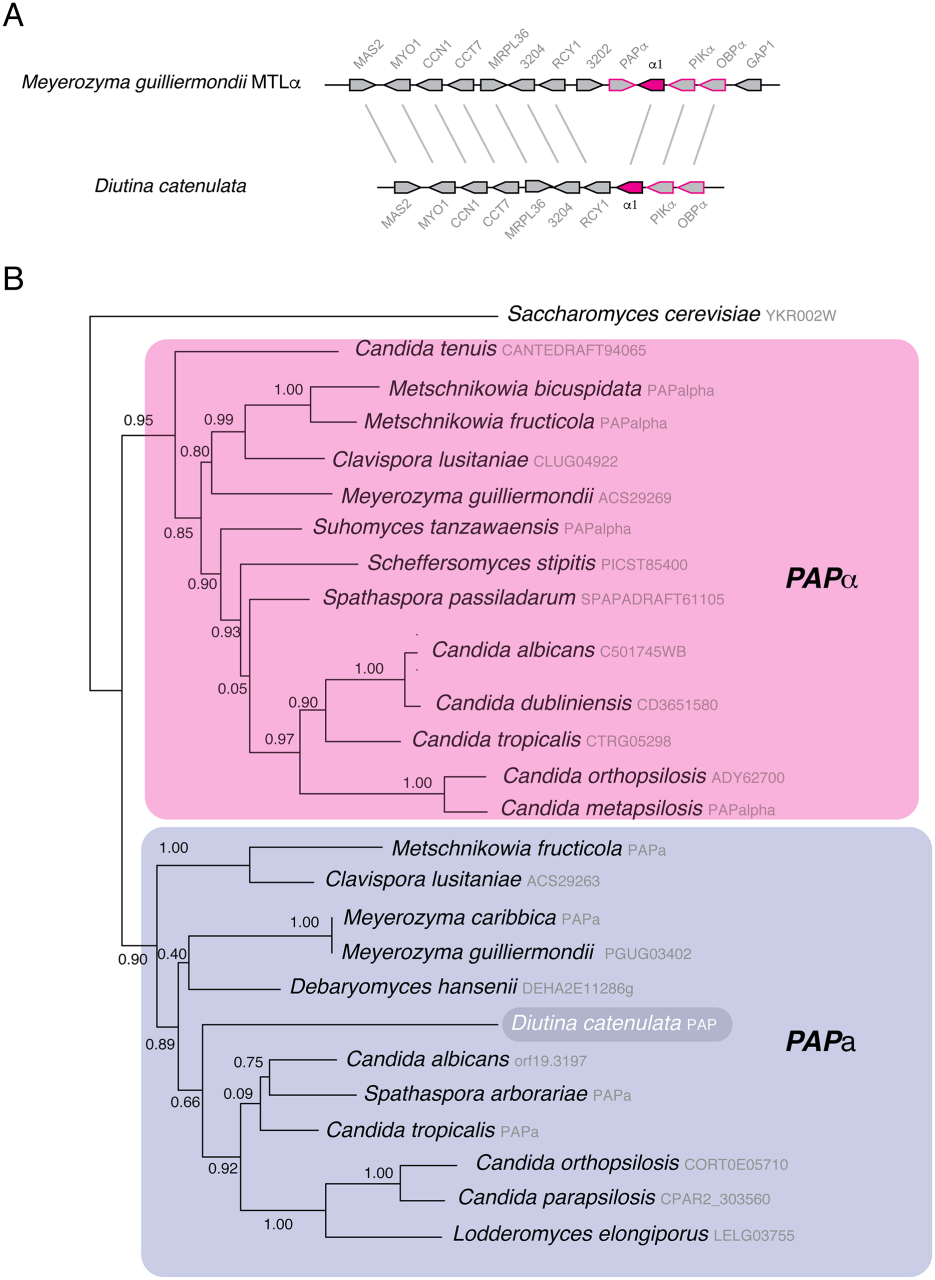
The Mating Type-like Locus in *D*. *catenulata*. A. Gene order around *MTL*α in *M*. *guilliermondii* and *D*. *catenulata*. Orthologous genes are connected with gray lines. Mating-type genes are filled in pink, and other genes associated with the *MTL* are edged in pink. The assembly of the *D*. *catenulata* contig stops at *OBP*. B. Phylogenetic relationship of *PAP*α and *PAP***a** from the indicated species from the Debaryomycetaceae/Metschnikowiaceae clade. The *PAP* protein from *D*. *catenulata* (which is not found at the *MTL* locus) is more closely related to *PAP***a** than to *PAP*α alleles. Alignments and phylogenetic trees were constructed using PhyML in SeaView [39].

Many species within the Lodderomyces clade are asexual, or at best undergo a parasexual cycle [55,56]. Mating and meiosis have however been described in several species outside the Lodderomyces clade, including *Debaryomyces hansenii* [57] and *Metschnikowia* species [43]. In both parasexual and fully sexual species, cell type is determined by alleles at the Mating-Type Like (*MTL*) loci, or idiomorphs. Transcriptional regulation by α1 and α2 (at the *MTL*α locus) or **a**1 and **a**2 (at the *MTL***a** locus) controls cell identity. However, the *MTL* loci also contain idiomorph-specific versions of *PAP* [poly(A) polymerase], *PIK* (phosphoinositol kinase), and *OBP* (oxysterol binding protein), which have no known role in mating [58]. Analysis of the likely *MTL* idiomorph of *D*. *catenulata* shows that it is very similar to *MTL*α of *M*. *guilliermondii* (Fig 3A). *OBP*, *PIK* and α1 genes are present, in the same order and in the same syntenic position as in *M*. *guilliermondii*. There is no obvious α2 sequence, but this has gene also been lost from *M*. *guilliermondii* and from several related species [55,56,59,60].

*orf19*.*3202*, a gene whose function is unknown, but which does not appear to be essential for viability in *C*. *albicans* [61], is also not present at *MTL* or elsewhere in the *D*. *catenulata* assembly. Surprisingly, there is also no *PAP*α sequence at the *D*. *catenulata MTL*. To eliminate the possibility of a misassembly at *MTL*, the region between *PIK*α and *RCY1* was amplified from the two isolates of *D*. *catenulata*. Sanger sequencing confirmed the loss of *orf19*.*3202* and *PAP* in both isolates (S2 File). BLAST analysis identified a *PAP* gene on a different contig in the WY3-10-4 genome. However, phylogenetic reconstruction shows that this is a *PAP***a**, rather than a *PAP*α, allele (Fig 3B). The simplest interpretation is that *D*. *catenulata* has a heterothallic structure at *MTL*, and that mating may occur between *MTL***a** isolates (currently uncharacterised) and *MTL*α isolates (like the sequenced strain). *PAP***a** may have become separated from *MTL***a** by a species-specific rearrangement.

## Conclusion

We used genomic analysis of tRNA complement, coding potential and phylogenetic analyses to address an outstanding question regarding the evolutionary relationship of *D*. *catenulata* to other yeast species in the Saccharomycotina. Our results categorically place *D*. *catenulata* in the Debaryomycetaceae/Metschnikowiaceae, or CTG-Ser clade, as an outgroup of the Lodderomyces clade.

## Supporting information

S1 Fig Supertree reconstruction of *D*. *catenulata* phylogeny. Heuristic Bayesian supertree reconstruction of 42 species based on 3,826 single-copy gene phylogenies was performed as described in Methods. The consensus phylogeny was visualized using iTOL. Clade colors are as described in Fig 1.
(PDF)

S1 File BLAST-based predictions of codon usage in *D*. *catenulata* isolate WY3-10-4. (XLS)

S2 File Sequence between *PIK1* and *RCY1*. (DOCX)

## References

[1] Delavenne E, Mounier J, Asmani K, Jany JL, Barbier G, Le Blay G (2011) Fungal diversity in cow, goat and ewe milk. Int J Food Microbiol 151: 247–251. doi: 10.1016/j.ijfoodmicro.2011.08.029 21944758 10.1016/j.ijfoodmicro.2011.08.029

[2] Gkatzionis K, Yunita D, Linforth RS, Dickinson M, Dodd CE (2014) Diversity and activities of yeasts from different parts of a Stilton cheese. Int J Food Microbiol 177: 109–116. doi: 10.1016/j.ijfoodmicro.2014.02.016 24631634 10.1016/j.ijfoodmicro.2014.02.016

[3] Facchin S, Barbosa AC, Carmo LS, Silva MC, Oliveira AL, Morais PB, et al (2013) Yeasts and hygienic-sanitary microbial indicators in water buffalo mozzarella produced and commercialized in Minas Gerais, Brazil. Braz J Microbiol 44: 701–707. 24516436 10.1590/s1517-83822013000300006 PMC3910177

[4] Cogan TM, Goerges S, Gelsomino R, Larpin S, Hohenegger M, Bora N, et al (2014) Biodiversity of the surface microbial consortia from Limburger, Reblochon, Livarot, Tilsit, and Gubbeen cheeses. Microbiol Spectr 2: CM-0010-2012. 10.1128/microbiolspec.CM-0010-2012 26082119

[5] Santin R, Mattei AS, Waller SB, Madrid IM, Cleff MB, Xavier MO, et al (2013) Clinical and mycological analysis of dog’s oral cavity. Braz J Microbiol 44: 139–143. doi: 10.1590/S1517-83822013005000018 24159296 10.1590/S1517-83822013005000018 PMC3804190

[6] Subramanya SH, Sharan NK, Baral BP, Hamal D, Nayak N, Prakash PY, et al (2017) Diversity, in-vitro virulence traits and antifungal susceptibility pattern of gastrointestinal yeast flora of healthy poultry, *Gallus gallus* domesticus. BMC Microbiol 17: 113 doi: 10.1186/s12866-017-1024-4 28506251 10.1186/s12866-017-1024-4 PMC5433169

[7] Mendes JF, Albano AP, Coimbra MA, Ferreira GF, Goncalves CL, Nascente Pda S, et al (2014) Fungi isolated from the excreta of wild birds in screening centers in Pelotas, RS, Brazil. Rev Inst Med Trop Sao Paulo 56: 525–528. doi: 10.1590/S0036-46652014000600012 25351548 10.1590/S0036-46652014000600012 PMC4296874

[8] Brilhante RSN, Silva ALD, Monteiro FOB, Guedes GMM, Sales JA, Oliveira JS, et al (2017) Yeasts from Scarlet ibises (*Eudocimus ruber*): A focus on monitoring the antifungal susceptibility of *Candida famata* and closely related species. Med Mycol 55: 725–732. doi: 10.1093/mmy/myw144 28204651 10.1093/mmy/myw144

[9] Urubschurov V, Janczyk P, Pieper R, Souffrant WB (2008) Biological diversity of yeasts in the gastrointestinal tract of weaned piglets kept under different farm conditions. FEMS Yeast Res 8: 1349–1356. doi: 10.1111/j.1567-1364.2008.00444.x 19054135 10.1111/j.1567-1364.2008.00444.x

[10] Janke T, Schwaiger K, Ege M, Fahn C, von Mutius E, Bauer J, et al (2013) Analysis of the fungal flora in environmental dust samples by PCR-SSCP method. Curr Microbiol 67: 156–169. doi: 10.1007/s00284-013-0344-3 23475138 10.1007/s00284-013-0344-3

[11] de Almeida JM (2005) Yeast community survey in the Tagus estuary. FEMS Microbiol Ecol 53: 295–303. doi: 10.1016/j.femsec.2005.01.006 16329949 10.1016/j.femsec.2005.01.006

[12] Radosavljevic M, Koenig H, Letscher-Bru V, Waller J, Maloisel F, Lioure B, et al (1999) *Candida catenulata* fungemia in a cancer patient. J Clin Microbiol 37: 475–477. 9889248 10.1128/jcm.37.2.475-477.1999 PMC84348

[13] Sarbu I, Pelinescu D, Stoica I, Marutescu L, Vassu T (2013) Phenotypic profiles of virulence in different *Candida* species isolated from vulvovaginal infections. Roum Arch Microbiol Immunol 72: 225–233. 24923105

[14] Joo HS, Ndegwa PM, Shoda M, Phae CG (2008) Bioremediation of oil-contaminated soil using *Candida catenulata* and food waste. Environ Pollut 156: 891–896. doi: 10.1016/j.envpol.2008.05.026 18620787 10.1016/j.envpol.2008.05.026

[15] Lachance M-A, Boekhout T, Scorzetti G, Fell JW, Kurtzmann CP (2011) Candida Berkhout (1923) In: Kurtzman CP, Fell JW, Boekhout T, editors. The Yeasts, a Taxonomic Study. 5th ed Amsterdam: Elsevier pp. 987–1278.

[16] Paredes K, Sutton DA, Cano J, Fothergill AW, Lawhon SD, Zhang S, et al (2012) Molecular identification and antifungal susceptibility testing of clinical isolates of the *Candida rugosa* species complex and proposal of the new species *Candida neorugosa*. J Clin Microbiol 50: 2397–2403. doi: 10.1128/JCM.00688-12 22553236 10.1128/JCM.00688-12 PMC3405575

[17] Chaves GM, Tercarioli GR, Padovan AC, Rosas RC, Ferreira RC, Melo AS, et al (2013) *Candida mesorugosa* sp. nov., a novel yeast species similar to *Candida rugosa*, isolated from a tertiary hospital in Brazil. Med Mycol 51: 231–242. doi: 10.3109/13693786.2012.710345 22928924 10.3109/13693786.2012.710345

[18] Khunnamwong P, Lertwattanasakul N, Jindamorakot S, Limtong S, Lachance MA (2015) Description of Diutina gen. nov., Diutina siamensis, f.a. sp. nov., and reassignment of Candida catenulata, Candida mesorugosa, Candida neorugosa, Candida pseudorugosa, Candida ranongensis, Candida rugosa and Candida scorzettiae to the genus Diutina. Int J Syst Evol Microbiol 65: 4701–4709. doi: 10.1099/ijsem.0.000634 26410375 10.1099/ijsem.0.000634

[19] Daniel HM, Lachance MA, Kurtzman CP (2014) On the reclassification of species assigned to *Candida* and other anamorphic ascomycetous yeast genera based on phylogenetic circumscription. Antonie Van Leeuwenhoek 106: 67–84. doi: 10.1007/s10482-014-0170-z 24748333 10.1007/s10482-014-0170-z

[20] Sylvester K, Wang QM, James B, Mendez R, Hulfachor AB, Hittinger CT (2015) Temperature and host preferences drive the diversification of *Saccharomyces* and other yeasts: a survey and the discovery of eight new yeast species. FEMS Yeast Res 15. 10.1093/femsyr/fov002 25743785

[21] White TJ, Bruns T, Lee S, Taylor J (1990) Amplification and direct sequencing of fungal ribosomal RNA genes for phylogenetics PCR protocols:A guide to Methods and Applications: Acadmeic Press, Inc pp. 315–322.

[22] Jiang H, Lei R, Ding SW, Zhu S (2014) Skewer: a fast and accurate adapter trimmer for next-generation sequencing paired-end reads. BMC Bioinformatics 15: 182 doi: 10.1186/1471-2105-15-182 24925680 10.1186/1471-2105-15-182 PMC4074385

[23] Bankevich A, Nurk S, Antipov D, Gurevich AA, Dvorkin M, Kulikov AS, et al (2012) SPAdes: a new genome assembly algorithm and its applications to single-cell sequencing. J Comput Biol 19: 455–477. doi: 10.1089/cmb.2012.0021 22506599 10.1089/cmb.2012.0021 PMC3342519

[24] Li H, Durbin R (2009) Fast and accurate short read alignment with Burrows-Wheeler transform. Bioinformatics 25: 1754–1760. doi: 10.1093/bioinformatics/btp324 19451168 10.1093/bioinformatics/btp324 PMC2705234

[25] McKenna A, Hanna M, Banks E, Sivachenko A, Cibulskis K, Kernytsky A, et al (2010) The Genome Analysis Toolkit: a MapReduce framework for analyzing next-generation DNA sequencing data. Genome Res 20: 1297–1303. doi: 10.1101/gr.107524.110 20644199 10.1101/gr.107524.110 PMC2928508

[26] Stanke M, Steinkamp R, Waack S, Morgenstern B (2004) AUGUSTUS: a web server for gene finding in eukaryotes. Nucleic Acids Res 32: W309–312. doi: 10.1093/nar/gkh379 15215400 10.1093/nar/gkh379 PMC441517

[27] Fitzpatrick DA, Logue ME, Stajich JE, Butler G (2006) A fungal phylogeny based on 42 complete genomes derived from supertree and combined gene analysis. BMC Evol Biol 6: 99 doi: 10.1186/1471-2148-6-99 17121679 10.1186/1471-2148-6-99 PMC1679813

[28] McCarthy CGP, Fitzpatrick DA (2017) Multiple approaches to phylogenomic reconstruction of the fungal Kingdom. Adv Genet 100: 211–266. doi: 10.1016/bs.adgen.2017.09.006 29153401 10.1016/bs.adgen.2017.09.006

[29] Edgar RC (2004) MUSCLE: a multiple sequence alignment method with reduced time and space complexity. BMC Bioinformatics 5: 113 doi: 10.1186/1471-2105-5-113 15318951 10.1186/1471-2105-5-113 PMC517706

[30] Castresana J (2000) Selection of conserved blocks from multiple alignments for their use in phylogenetic analysis. Mol Biol Evol 17: 540–552. doi: 10.1093/oxfordjournals.molbev.a026334 10742046 10.1093/oxfordjournals.molbev.a026334

[31] Darriba D, Taboada GL, Doallo R, Posada D (2011) ProtTest 3: fast selection of best-fit models of protein evolution. Bioinformatics 27: 1164–1165. doi: 10.1093/bioinformatics/btr088 21335321 10.1093/bioinformatics/btr088 PMC5215816

[32] Guindon S, Dufayard JF, Lefort V, Anisimova M, Hordijk W, Gascuel O (2010) New algorithms and methods to estimate maximum-likelihood phylogenies: assessing the performance of PhyML 3.0. Syst Biol 59: 307–321. doi: 10.1093/sysbio/syq010 20525638 10.1093/sysbio/syq010

[33] Akanni WA, Wilkinson M, Creevey CJ, Foster PG, Pisani D (2015) Implementing and testing Bayesian and maximum-likelihood supertree methods in phylogenetics. R Soc Open Sci 2: 140436 doi: 10.1098/rsos.140436 26361544 10.1098/rsos.140436 PMC4555849

[34] Letunic I, Bork P (2016) Interactive tree of life (iTOL) v3: an online tool for the display and annotation of phylogenetic and other trees. Nucleic Acids Res 44: W242–245. doi: 10.1093/nar/gkw290 27095192 10.1093/nar/gkw290 PMC4987883

[35] Kuck P, Meusemann K (2010) FASconCAT: Convenient handling of data matrices. Mol Phylogenet Evol 56: 1115–1118. doi: 10.1016/j.ympev.2010.04.024 20416383 10.1016/j.ympev.2010.04.024

[36] Swofford LD (2002) PAUP*: phylogenetic analysis using parsimony (* and other methods)version 4.0 beta. Sinauer Associates, Sunderland.

[37] Lartillot N, Philippe H (2004) A Bayesian mixture model for across-site heterogeneities in the amino-acid replacement process. Mol Biol Evol 21: 1095–1109. doi: 10.1093/molbev/msh112 15014145 10.1093/molbev/msh112

[38] Lartillot N, Rodrigue N, Stubbs D, Richer J (2013) PhyloBayes MPI: phylogenetic reconstruction with infinite mixtures of profiles in a parallel environment. Syst Biol 62: 611–615. doi: 10.1093/sysbio/syt022 23564032 10.1093/sysbio/syt022

[39] Gouy M, Guindon S, Gascuel O (2010) SeaView version 4: A multiplatform graphical user interface for sequence alignment and phylogenetic tree building. Mol Biol Evol 27: 221–224. doi: 10.1093/molbev/msp259 19854763 10.1093/molbev/msp259

[40] Byrne KP, Wolfe KH (2005) The Yeast Gene Order Browser: Combining curated homology and syntenic context reveals gene fate in polyploid species. Genome Res 15: 1456–1461. doi: 10.1101/gr.3672305 16169922 10.1101/gr.3672305 PMC1240090

[41] Byrne KP, Wolfe KH (2006) Visualizing syntenic relationships among the hemiascomycetes with the Yeast Gene Order Browser. Nucleic Acids Res 34: D452–455. doi: 10.1093/nar/gkj041 16381909 10.1093/nar/gkj041 PMC1347404

[42] Groenewald M, Smith MT (2010) Re-examination of strains formerly assigned to Hyphopichia burtonii, the phylogeny of the genus Hyphopichia, and the description of Hyphopichia pseudoburtonii sp. nov. Int J Syst Evol Microbiol 60: 2675–2680. doi: 10.1099/ijs.0.018580-0 19965989 10.1099/ijs.0.018580-0

[43] Lachance MA (2016) *Metschnikowia*: half tetrads, a regicide and the fountain of youth. Yeast 33: 563–574. 10.1002/yea.3208 27599462

[44] Lachance MA, Hurtado E, Hsiang T (2016) A stable phylogeny of the large-spored *Metschnikowia* clade. Yeast 33: 261–275. doi: 10.1002/yea.3163 27019191 10.1002/yea.3163

[45] Lachance MA, Miranda M, Miller MW, Phaff HJ (1976) Dehiscence and active spore release in pathogenic strains of the yeast *Metschnikowia bicuspidata* var. australis: possible predatory implication. Can J Microbiol 22: 1756–1761. 1009503 10.1139/m76-259

[46] Hittinger CT, Rokas A, Bai FY, Boekhout T, Goncalves P, Jeffries TW, et al (2015) Genomics and the making of yeast biodiversity. Curr Opin Genet Dev 35: 100–109. doi: 10.1016/j.gde.2015.10.008 26649756 10.1016/j.gde.2015.10.008 PMC4771062

[47] Shen XX, Zhou X, Kominek J, Kurtzman CP, Hittinger CT, Rokas A (2016) Reconstructing the backbone of the Saccharomycotina yeast phylogeny using genome-scale data. G3 (Bethesda) 6: 3927–3939. 27672114 10.1534/g3.116.034744 PMC5144963

[48] Morais CG, Batista TM, Kominek J, Borelli BM, Furtado C, Moreira RG, et al (2017) *Spathaspora boniae* sp. nov., a D-xylose-fermenting species in the *Candida albicans*/*Lodderomyces* clade. Int J Syst Evol Microbiol 67: 3798–3805. doi: 10.1099/ijsem.0.002186 28884677 10.1099/ijsem.0.002186

[49] Santos MA, Gomes AC, Santos MC, Carreto LC, Moura GR (2011) The genetic code of the fungal CTG clade. Comptes rendus biologies 334: 607–611. doi: 10.1016/j.crvi.2011.05.008 21819941 10.1016/j.crvi.2011.05.008

[50] Riley R, Haridas S, Wolfe KH, Lopes MR, Hittinger CT, Goker M, et al (2016) Comparative genomics of biotechnologically important yeasts. Proc Natl Acad Sci U S A 113: 9882–9887. doi: 10.1073/pnas.1603941113 27535936 10.1073/pnas.1603941113 PMC5024638

[51] Muhlhausen S, Findeisen P, Plessmann U, Urlaub H, Kollmar M (2016) A novel nuclear genetic code alteration in yeasts and the evolution of codon reassignment in eukaryotes. Genome Res 26: 945–955. doi: 10.1101/gr.200931.115 27197221 10.1101/gr.200931.115 PMC4937558

[52] Yokogawa T, Suzuki T, Ueda T, Mori M, Ohama T, Kuchino Y, et al (1992) Serine tRNA complementary to the nonuniversal serine codon CUG in *Candida cylindracea*: evolutionary implications. Proc Natl Acad Sci U S A 89: 7408–7411. 1502151 10.1073/pnas.89.16.7408 PMC49719

[53] Lowe TM, Eddy SR (1997) tRNAscan-SE: a program for improved detection of transfer RNA genes in genomic sequence. Nucleic Acids Res 25: 955–964. 9023104 10.1093/nar/25.5.955 PMC146525

[54] Santos MA, Ueda T, Watanabe K, Tuite MF (1997) The non-standard genetic code of *Candida* spp.: an evolving genetic code or a novel mechanism for adaptation? Mol Microbiol 26: 423–431. 9402014 10.1046/j.1365-2958.1997.5891961.x

[55] Bennett RJ, Turgeon BG (2016) Fungal Sex: The Ascomycota In: H J, H B, C P, S E, J T et al, editors. The Fungal Kingdom. Washington, DC: ASM Press pp. 117–145.

[56] Wolfe KH, Butler G (2017) Evolution of mating in the Saccharomycotina. Annu Rev Microbiol 71: 197–214. doi: 10.1146/annurev-micro-090816-093403 28657889 10.1146/annurev-micro-090816-093403

[57] van der Walt JP, Taylor MB, Liebenberg NV (1977) Ploidy, ascus formation and recombination in *Torulaspora* (*Debaryomyces*) *hansenii*. Antonie Van Leeuwenhoek 43: 205–218. 596863 10.1007/BF00395675

[58] Srikantha T, Daniels KJ, Pujol C, Sahni N, Yi S, Soll DR (2012) Nonsex genes in the mating type locus of *Candida albicans* play roles in a/alpha biofilm formation, including impermeability and fluconazole resistance. PLoS pathogens 8: e1002476 doi: 10.1371/journal.ppat.1002476 22253594 10.1371/journal.ppat.1002476 PMC3257300

[59] Butler G (2007) The evolution of *MAT*: the ascomycetes In: Heitman J, Kronstad JW, Taylor JW, Casselton LA, editors. Sex in Fungi. Washington, D.C.: ASM Press pp. 3–18.

[60] Butler G (2010) Fungal sex and pathogenesis. Clin Microbiol Rev 23: 140–159. doi: 10.1128/CMR.00053-09 20065328 10.1128/CMR.00053-09 PMC2806657

[61] Nobile CJ, Bruno VM, Richard ML, Davis DA, Mitchell AP (2003) Genetic control of chlamydospore formation in *Candida albicans*. Microbiology 149: 3629–3637. doi: 10.1099/mic.0.26640-0 14663094 10.1099/mic.0.26640-0

