# Supplementary material for: Genome analysis of the yeast Diutina catenulata, a member of the Debaryomycetaceae/Metschnikowiaceae (CTG-Ser) clade
Source: PLoS One. 2018 Jun 26;13(6):e0198957. doi: 10.1371/journal.pone.0198957 (PMC6019693; doi:10.1371/journal.pone.0198957)
Supplement: S1 Fig — Heuristic Bayesian supertree reconstruction of 42 species based on 3,826 single-copy gene phylogenies was performed as described in Methods. The consensus phylogeny was visualized using iTOL. Clade colors are as described in Fig 1. (PDF) [file pone.0198957.s001.pdf]

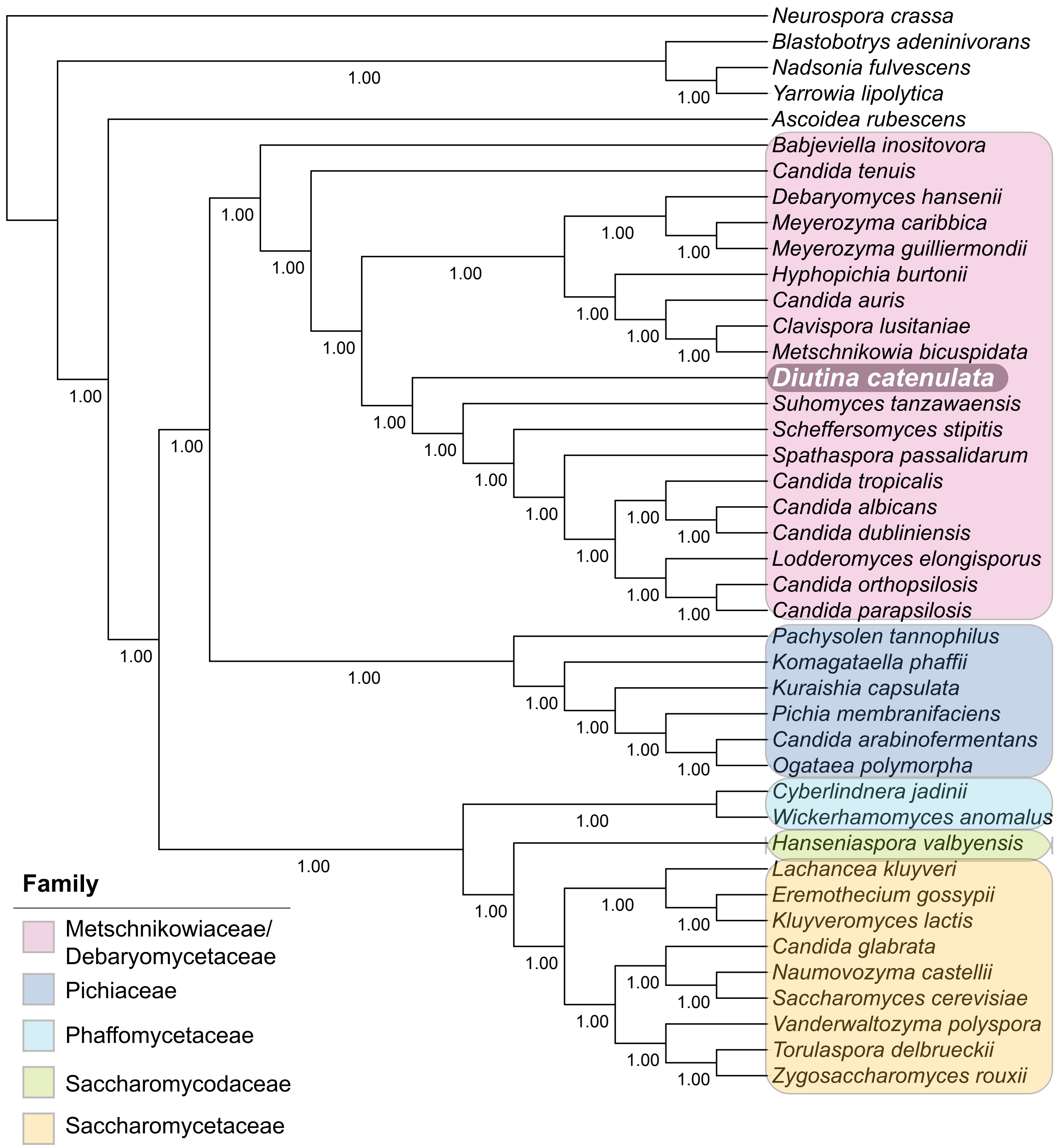

S1 Fig. Supertree reconstruction of *D. catenulata* phlogeny.

Heuristic Bayesian supertree reconstruction of 42 species based on 3,826 single-copy gene phylogenies was performed as described in Methods. The consensus phylogeny was visualized using iTOL. Clade colors are as described in Fig. 1.
