# Supplementary material for: Genome analysis of the yeast Diutina catenulata, a member of the Debaryomycetaceae/Metschnikowiaceae (CTG-Ser) clade
Source: PLoS One. 2018 Jun 26;13(6):e0198957. doi: 10.1371/journal.pone.0198957 (PMC6019693; doi:10.1371/journal.pone.0198957)
Supplement: S2 File — (DOCX) [file pone.0198957.s003.docx]

File S2. Sequence between *PIK1* and *RCY1*

>WY3-10-4_MTL

gcggttgcagatggagttgctggacgacgagtccgacgtgtttgtcgacgggctcattgc

caagagcgcggggtcgatgtacacccggttgtacgaccaatttcagatggtaacgcaagg

tatttactactagacaattttcgcatagcattagccacttattttcatctagtcatacat

gcgtgttgcgtacagaaaataacttagaactcacttataaccgaaggtggcgaactaata

cacgagaaattcagtaaggaggtcatggcttttctcgaaatacgacttgaatttgaacat

atcttgagctcgtttggcatgtatatagcttgccatcgaatctcgattagtgctccaatt

tcatccatgataccgtgcaatctgtatgtccgagataataaaacatcgtcgtcacaccga

attccgctcttataagtccatgaaatccgaagaacctgaaacaaaattataattaaatgg

caacgacacgatctctgtctattgcccaatcaaagcgaaaccctaagagggcatctaaaa

ggaaatccaaaaaccccaatattaccattttccgaatcgacaaacccgatcacaccgata

ccaaagagaagaccacaaattttcagatacaaccaagaactgagggtagcagtggagtgt

ttgttggccgtccatttgaattcaaaccaaagtttgacgatgcatggatcaaatatttga

tcaatttgaataacaatataccgccaggaaggggcggtcggcatcgttcgagattcaata

agaaagccaaaattaatgggtttctcgccttcaggatcttcctatctaaagatattaaga

gttatgatgttcaaagcagcgagctctccacgatggtaagtaacttttggaattccttac

cagaggaacaaaaggaaggctggagtaagctcgcatcaatttatctggaacaggttaaag

gcatggaacccaagccatccttcaacagttggtacctcaactttcgcgattcgaatgctc

aacaatggagtagaactcataaagagcttgcacagggcttacataaagatacttcgaaac

caagttaagctaggtttgaggagaacttggtgcatgggacagtttcatttctcatagggg

ccttcgtagttccacaatcttgtggcgatcgcgccgtacataaggtaaatatagaatcca

ggcaccgtggtgcacaaccctgcatatagaccgtgcccgtcacaagcgccgttacgtgga

gggcttacagaaacgttgacaccgcatgctaccatggcccaggtcatttggaacaacgac

atcgatgtctacaaccacgatgtcatgcccg

>UCD133_MTL

gttgagcagcggttgcagatggagttgctggacgacgagtccgacgtgtttgtcgacggg

ctcattgccaagagcgcggggtcgatgtacacccggttgtacgaccaatttcagatggta

acgcaaggtatttactactagacaattttcgcatagcattagccacttattttcatctag

tcatacatgcgtgttgcgtacagaaaataacttagaactcacttataaccgaaggtggcg

aactaatacacgagaaattcagtaaggaggtcatggcttttctcgaaatacgacttgaat

ttgaacatatcttgagctcgtttggcatgtatatagcttgccatcgaatctcgattagtg

ctccaatttcatccatgataccgtgcaatctgtatgtccgagataataaaacatcgtcgt

cacaccgaattccgctcttataagtccatgaaatccgaagaacctgaaacaaaattataa

ttaaatggcaacgacacgatctctgtctattgcccaatcaaagcgaaaccctaagagggc

atctaaaaggaaatccaaaaaccccaatattaccattttccgaatcgacaaacccgatca

caccgataccaaagagaagaccacaaattttcagatacaaccaagaactgagggtagcag

tggagtgtttgttggccgtccatttgaattcaaaccaaagtttgacgatgcatggatcaa

atatttgatcaatttgaataacaatataccgccaggaaggggcggtcggcatcgttcgag

attcaataagaaagccaaaattaatgggtttctcgccttcaggatcttcctatctaaaga

tattaagagttatgatgttcaaagcagcgagctctccacgatggtaagtaacttttggaa

ttccttaccagaggaacaaaaggaaggctggagtaagctcgcatcaatttatctggaaca

ggttaaaggcatggaacccaagccatccttcaacagttggtacctcaactttcgcgattc

gaatgctcaacaatggagtagaactcataaagagcttgcacagggcttacataaagatac

ttcgaaaccaagttaagctaggtttgaggagaacttggtgcatgggacagtttcatttct

cataggggccttcgtagttccacaatcttgtggcgatcgcgccgtacataaggtaaatat

agaatccaggcaccgtggtgcacaaccctgcatatagaccgtgcccgtcacaagcgccgt

tacgtggagggcttacagaaacgttgacaccgcatgctaccatggcccaggtcatttgga

acaacgacatcgatgtctacaaccacgatg
